# Interface treatment using amorphous-carbon and its applications

**DOI:** 10.1038/s41598-020-61141-9

**Published:** 2020-03-05

**Authors:** Myung Sik Choi, Han Gil Na, Jae Hoon Bang, Sun-Woo Choi, Sang Sub Kim, Kyu Hyoung Lee, Hyoun Woo Kim, Changhyun Jin

**Affiliations:** 1 Division of Materials Science and Engineering, Hanyang University, Seoul, 04763 Republic of Korea; 2 Department of Materials Science and Engineering, Kangwon National University, Samcheok, 25913 Republic of Korea; 3 Department of Materials Science and Engineering, Inha University, Incheon, 402-751 Republic of Korea; 4 Department of Materials Science and Engineering, Yonsei University, Seoul, 03722 Republic of Korea

**Keywords:** Chemical synthesis, Nanoscale materials

## Abstract

Breakthrough process technologies have been introduced that can increase the chemical sensitivity of an interface at which reactions occur without significantly altering the physico-chemical properties of the material. Such an interfacial treatment method is based on amorphous-carbon as a base so that fluids can be deposited, and the desired thickness and quality of the deposition can be ensured irrespective of the interface state of the material. In addition, side effects such as diffusion and decreasing strength at the interface can be avoided. This is simpler than existing vacuum-based deposition technology and it has an unmatched industrial advantage in terms of economics, speed, accuracy, reliability, accessibility, and convenience. In particular, this amorphous-carbon interface treatment technology has been demonstrated to improve gas-sensing characteristics of NO_2_ at room temperature.

## Introduction

Techniques such as electroplating^1^, electroless plating^2^, anodisation^3^, chemical treatment^4^, plasma surface treatment^5^, and dry coating^6^, in which one or more thin layers are overlayed on a material, protect the surface of the material^7^, increase its strength^8^, and add functionality^9^. And most of these technologies are limited to bulk materials, rather than nanomaterials, such as automotive-, machine-, tool-, and mold-materials^10–13^, based on progress in science/engineering. Also, as the manufacture of thin films^14^ and core-shell structures^15^ by vacuum deposition^16^ and vacuum coating^17^ is gaining momentum, particularly for home appliances, electronic components, optical components, and biomaterials^18–20^, sub-factors such as clean technology, evaporation source selectivity, product-specific process technologies, and functional deposition materials, became one of the main technologies. However, in order to realise the manufacture of complete thin films/coatings such as natural simulation^21,22^, engineered structure implementation using hybrid processes^23,24^, high-speed deposition^25^, and cost-saving technology^26^, many problems have to be overcome, as follows.

1. The state of the surface of a material may significantly influence the adhesion of the thin film/coating and the characteristics and life of the product; hence, an extra process must be performed to remove the impurities and the oxide film^27^.
2. Although the process itself is simple, the evaporation technique is unidirectional. Therefore, step coverage cannot be avoided^28^.
3. Deposition via metal-organic chemical vapour deposition (MOCVD) has the disadvantage that growth rate and impurity doping are possible only with substrate temperature and gas flow rate, but using hazardous materials and high equipment/source cost^29^.
4. Deposition via atomic layer deposition (ALD) has the advantages of thin film deposition and low impurity content on an atomic level, but the deposition rate is limited by the ALD mechanism^30^.
5. Sputtering can be used to control certain aspects of thin films of difficult materials, such as high melting points, but it requires large amounts of gas for ionisation and its efficiency is poor^31^.

As described above, an advantage in one process can be a disadvantage in the other; hence, based on many considerations, the best process can be determined depending on which factors one focuses on. Processes or deposition/coating techniques with many advantages at the same time have not yet been introduced or tested. In addition, when a heterogeneous material is deposited on an existing product or substrate to form a new double layer, the properties of the individual materials may be lost owing to interdiffusion^32^ between the two materials. Even if the interdiffusion is controlled, because the arrangement of irregular atoms at the bonding interface cannot be avoided, the strength of the irregular atoms must be lower than that of the matrix. As such, the bonding interface of the heterogeneous materials cannot but react sensitively, unlike the inner parts of the material. If so, is there no means of enhancing the properties of existing materials while increasing the adsorption strength during adsorption to other materials? If one considers several prerequisites to take only the selfish advantage, the following conditions must be met. First, in order to increase the adsorption strength regardless of the state of the other interfaces of all products, the deposition material must have characteristics that enable it to cover the substrate like a fluid. Second, in order to increase the characteristics of the coated material, it is necessary to reduce the characteristics of the deposited material to a minimum. In order to satisfy these two conditions, during the deposition of the heterogeneous material (heterojunction), the properties of the surface (interface) of the coated material, must be prominently highlighted. Hence, we propose a simple interface treatment technique using amorphous-carbon (a-C), which is advantageous in terms of economics, speed, accuracy, and reliability, as described above. This technique can be used to realise powerful synthesis, deposition, and coating.

## Result and Discussion

### Morphologies and microstructures of a-C

**Figure 1:**
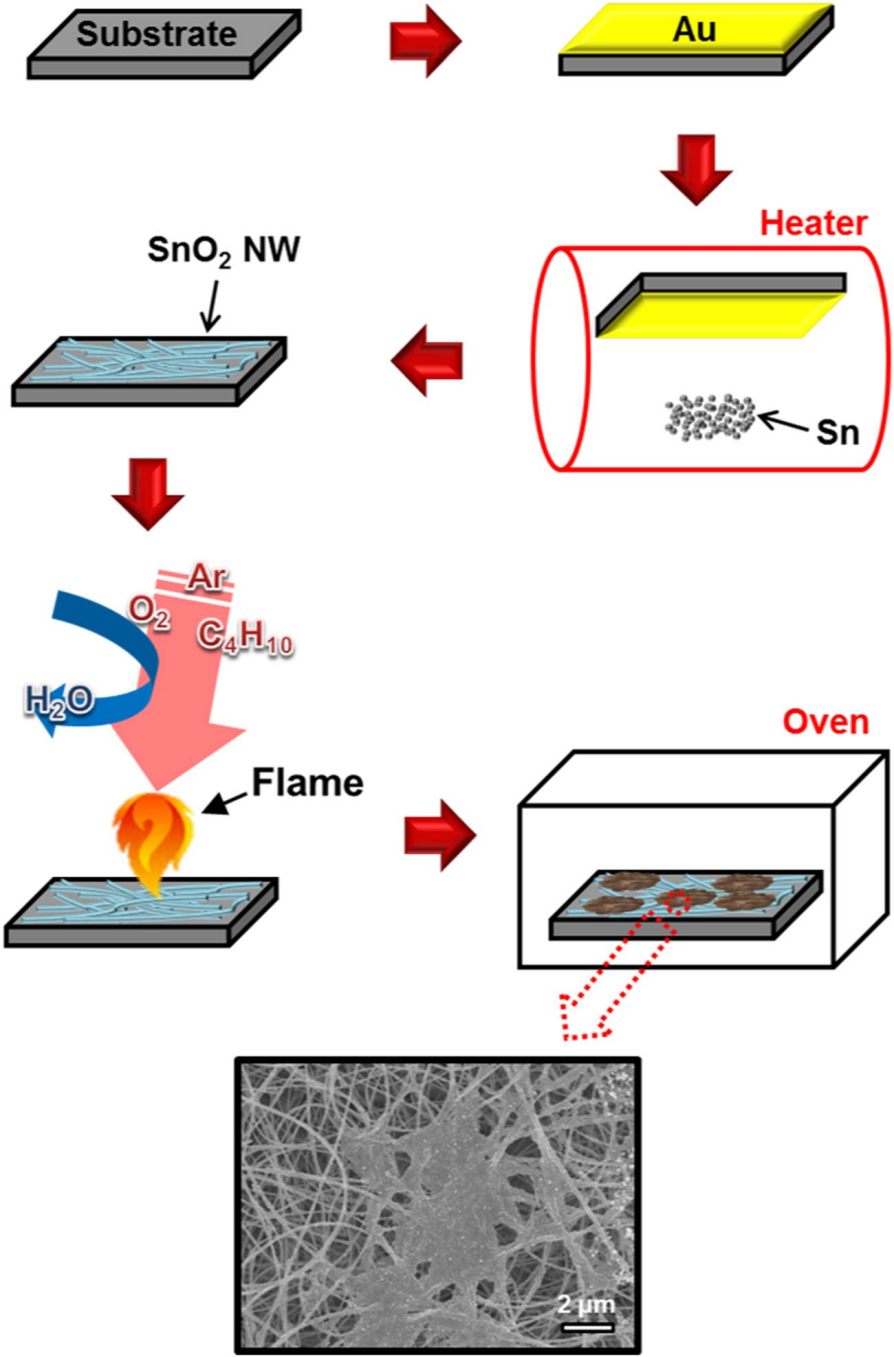
Synthesis of SnO_2_/a-C core-shell structure. SnO_2_ was formed via thermal evaporation of Sn powder using a Au catalyst on an alumina substrate, and the a-C thin film was obtained by applying a spark to water, which was drained and deposited on the SnO_2_ NWs.

**Figure 2:**
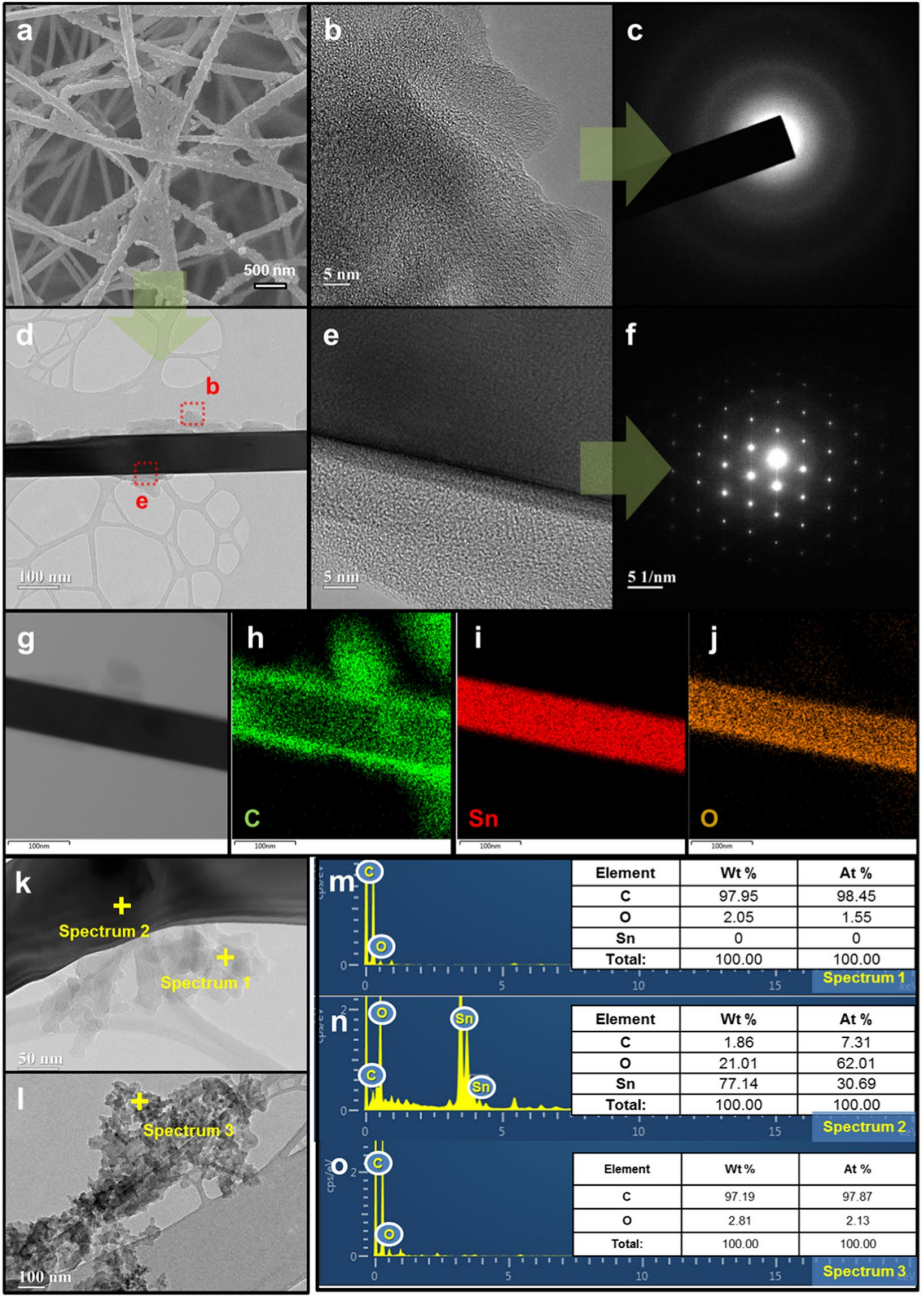
Morphology, crystallinity, and elemental composition of SnO_2_/a-C. (**a**) SEM image of SnO_2_ coated with a-C; (**b**) HRTEM image of a-C as the coating layer; (**c**) SAED pattern of a-C; (**d**) single SnO_2_/a-C core-shell structure; (**e**) HRTEM image of SnO_2_/a-C interface; (**f**) SAED pattern of mixture of monocrystalline SnO_2_ and a-C; (**g**) TEM image of SnO_2_/a-C before mapping; distribution of (**h**) C, (**i**) Sn, and (**j**) O in SnO_2_/a-C; (**k**) TEM image of SnO_2_/a-C interface; (**l**) TEM image of a-C; point EDX results of (**m**) a-C shell, (**n**) SnO_2_ core, and (**o**) a–C.

The conditions and preparation of a-C as a coating are described in the methods section and Fig. 1. Supplementary information (SI), Fig. S1 shows SEM images of cases where a-C is applied in the form of a thin film and a core-shell to an alumina substrate and SnO_2_ nanowires (NWs), respectively. In the case of the alumina substrate, it was observed that nanoparticles aggregated when a-C was deposited on the alumina surface. These a-C particles could be determined by adjusting the synthesis temperature or process time (the corresponding images are not shown). Then, the a-C particles can be considered as heterogeneous nucleation mechanisms rather than homogeneous nucleation mechanisms based on the grain boundaries of existing alumina substrates. In the case of the SnO_2_ NWs, the results show that this synthesis technique was applied successfully. SI, Figs. S1g,h indicate that an a-C structure can be formed in spaces between SnO_2_ NWs as well as on the surface of SnO_2_ NWs. The crystallisation characteristics of a-C microstructures that can be simultaneously applied to thin films and NWs of SnO_2_ (Fig. 2a) were confirmed by transmission electron microscopy (TEM; Fig. 2d,g,k,l), high-resolution TEM (HRTEM; Fig. 2b,e), selected-area electron diffraction (SAED; Fig. 2c,f) patterns, mapping (Figs. 2h–j), and point energy dispersive X-ray spectroscopy (EDX, Fig. 2m–o). HRTEM and SAED patterns were observed for each part (SnO_2_ core and a-C shell) of the SnO_2_ NWs to investigate the crystallinity of SnO_2_ NWs covered by a-C. It was found that single crystals of SnO_2_ were represented by spotty patterns and a-C was represented by hazy circles. In particular, as seen in Fig. 2f, the presence of blurred circles in the spotty pattern of SnO_2_ implies the possibility of the transfer of properties different from those of the existing materials without significantly affecting the original characteristics of the existing materials. The mapping results confirm that the a-C (Fig. 2h) uniformly covers the preformed SnO_2_ NWs (Fig. 2I,j). With regard to the distribution of carbon, the concentration at the surface of the SnO_2_ NWs is the greatest, which indicates the nature of the surface of the SnO_2_ NWs (or the nature of the interface between SnO_2_ and a-C) (Fig. 2h). The composition and properties of carbon were reaffirmed using point EDX (Fig. 2m–o). That is, the deposition layer, which can cover the surface of the material easily, similar to a fluid, is almost entirely carbon (Fig. 2m,o), most of which is present at the interface of the existing materials. This means that there is hardly any interdiffusion of atoms in the existing heterogeneous junctions. In summary, only the energy characteristics of the SnO_2_ interface at which all reactions occur are changed while the inherent characteristics of the SnO_2_ are maintained.

### Properties of SnO_2_/a-C core-shell interface

**Figure 3:**
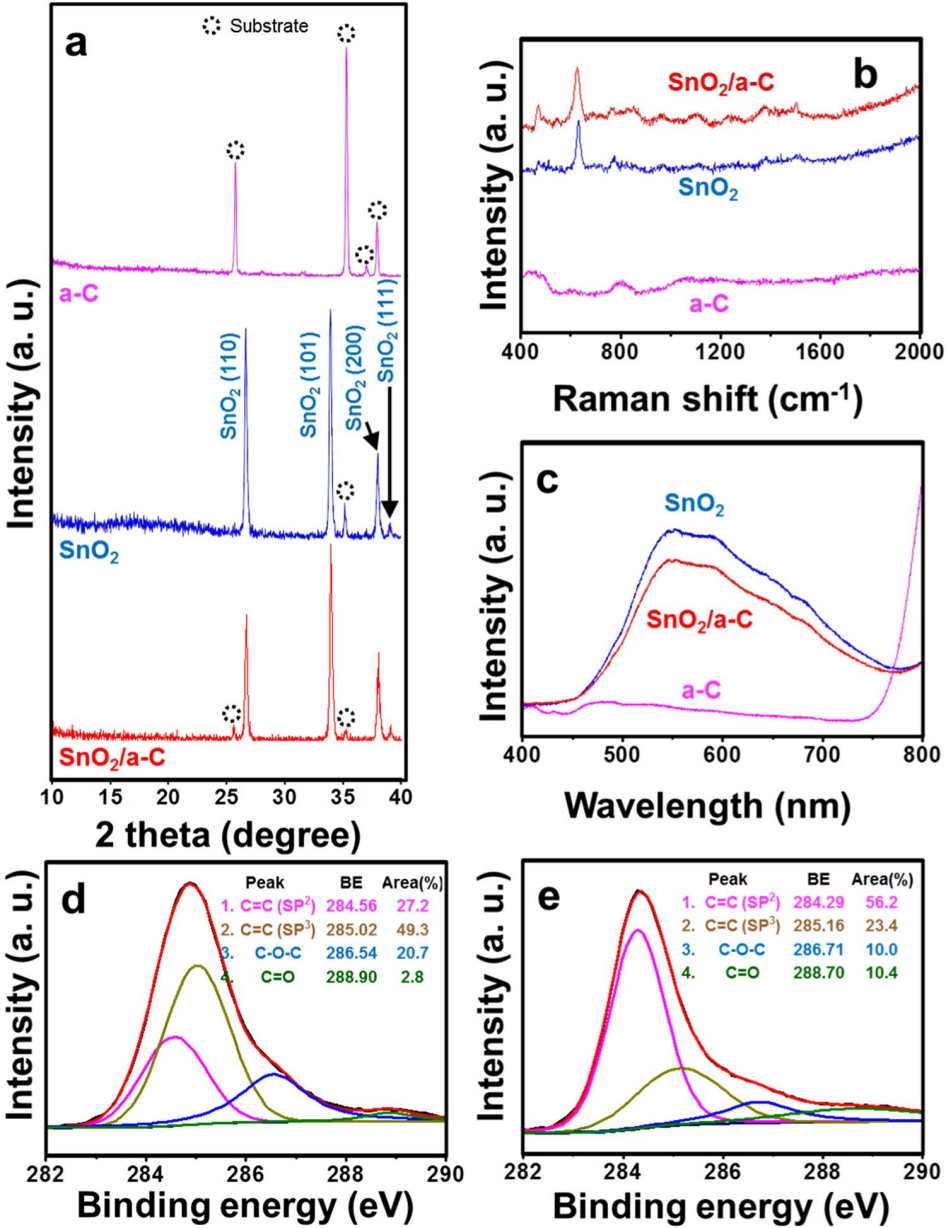
Analysis of chemical bonding of a-C, SnO_2_, and SnO_2_/a-C. (**a**) XRD results, (**b**) Raman spectra, and (**c**) PL of each sample; XPS results after deposition of a-C on (**d**) alumina substrate and (**e**) SnO_2_ NWs.

Figure 3 presents evidence index results that show the coating properties of the SnO_2_ cores and a-C shell structures. The XRD results (Fig. 3a) indicate that a-C does not exhibit its own crystalline properties and can affect only by bonding with other materials (SnO_2_). The XRD patterns for the deposition on the existing substrate (pink) and the coating on the SnO_2_ NWs (red) do not exhibit their own unique peaks. This tendency is also prominent in the results of the Raman (Fig. 3b) and PL (Fig. 3c) measurements. However, even if its own characteristics are not apparent, the effects of a-C cannot be ignored, as the existing peaks vary in intensity. This difference can clearly be observed in the XPS results of the a-C binding energies in the a-C thin film and SnO_2_/a-C shown in Fig. 3d,e, respectively. The binding originally exhibited by a-C is dominated by sp3 binding rather than sp2 binding (Fig. 3d). However, when a-C binds with SnO_2_, sp2 binding becomes more significant than sp3 binding, and the remaining C-O-C binding peak is significantly reduced (Fig. 3e). In other words, a-C does not have any effect on its own, but its combination with other materials can result in the creation of new properties at the interface.

### Energy exchange between materials

**Figure 4:**
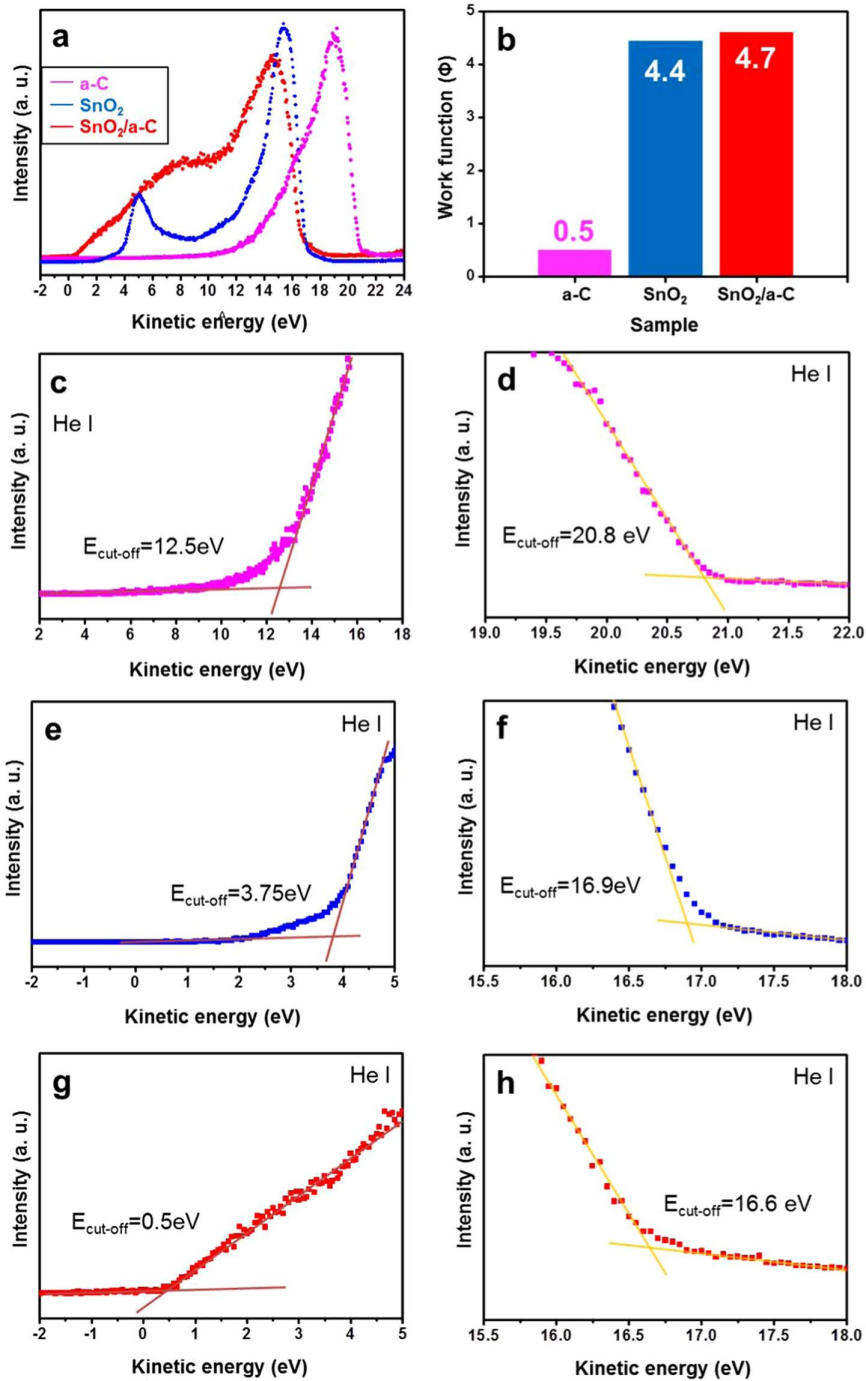
UPS results of a-C, SnO_2_, and SnO_2_/a-C. (**a**) UPS of a-C, crystalline SnO_2_, and SnO_2_/a-C core-shell; (**b**) work functions of a-C, SnO_2_, and SnO_2_/a-C; (**c**) valence band maximum of 12.5 eV and (**d**) energy_(cut-off)_ of 20.8 eV for work function of 0.5 eV in a-C (i.e., 21.2 eV_(incident energy)_ − 0.1 eV_(correction value)_); (**e**) valence band maximum of 3.75 eV and (**f**) energy_(cut-off)_ of 16.9 eV for work function of 4.4 eV in monocrystalline SnO_2_ (i.e., 21.2 eV_(incident energy)_ − 16.9 eV + 0.1 eV_(correction value)_).

**Figure 5:**
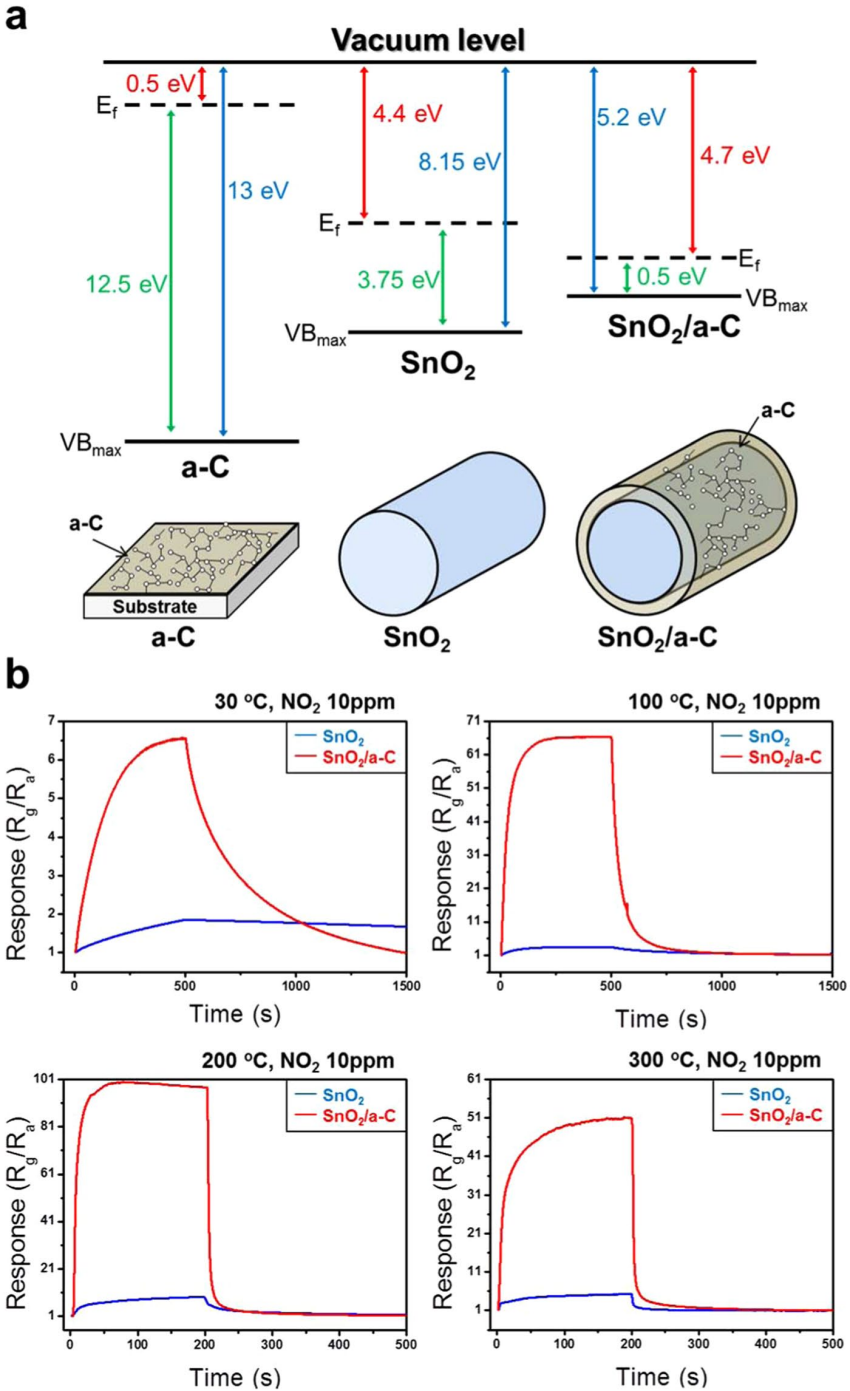
Comparison of energy states and 10 ppm NO_2_ gas-sensing performance for a-C, SnO_2_, SnO_2_/a-C. (**a**) Energy relationship between Fermi level, valence band maximum, and work function; (**b**) response at 30 °C, 100 °C, 200 °C, and 300 °C for SnO_2_ and SnO_2_/a-C core-shell structures.

As described above, although the physical and chemical properties of base materials can be changed or highlighted via deposition of a-C, there is still insufficient evidence to assert this conclusion. Therefore, ultraviolet photoelectron spectroscopy (UPS), which can be used to infer the mutual energy band by combining with the a-C, is used to clarify the differences in the valence band maximum and the work function of the preformed SnO_2_. Figure 4a presents the energy bands of a-C, SnO_2_, and SnO_2_/a-C. From the results of the incident energy and Fig. 4d,f,h, the work functions of a-C, SnO_2_, and SnO_2_/a-C are 0.5 eV, 4.4 eV, and 4.7, respectively, as shown in Fig. 4b. The difference between 4.4 eV and 4.7 eV can be assumed to be related to energy exchange before and after the deposition of a-C on SnO_2_. The changes in the valence band maximum of a-C, SnO_2_, and SnO_2_/a-C were 13 eV (12.5 eV V10.5 eV, Figs. 4b,c, and 5a), 8.15 eV (3.75 eV V34.4 eV, Figs. 4b,e and 5a), and 5.2 eV (0.5 eV V04.7 eV, Figs. 4b,g and 5a), respectively, on the basis of the vacuum level. This means that there is some energy interchange between SnO_2_ and a-C, which is a result of a completely new property based on the combination of SnO_2_ and a-C. To understand why this difference occurs and the exact role of a-C in it, let us consider all the possible predictions presented above by applying them directly to applications under different conditions with sensitive surface gas sensing. We must also understand how a-C works in combination with the existing materials. Therefore, in order to investigate the above predictions, we studied gas-sensing applications under different conditions because it is most sensitive to the surface. Based on this, the mechanism through which a-C combines with the existing material was deduced.

### Mechanism behind change in SnO_2_/a-C interface and gas sensing

Based on the UPS results (Fig. 4), the energy states for each material were deduced, as shown in Fig. 5a. The core-shell structure of SnO_2_/a-C had a valence band maximum of 5.2 eV and a Fermi level of 4.7 eV from the vacuum level. This energy structure exhibits superior gas-sensing characteristics with regard to process temperatures, responses, response times, and recovery times, compared to conventional gas sensing with bare SnO_2_ NWs^33–35^. This can be attributed to the a-C mechanism, which is optimised for the most prominent gas sensing, where the interfacial characteristics of the sample are more important than other factors during the gas measurement. In general, gas sensors are driven by activated carriers at the sample surface. However, the deposition of a-C on the SnO_2_ surface results in a lower energy barrier that can respond to gas sensing on the surface. Therefore, even if the bare SnO_2_ NWs do not exhibit gas-sensing characteristics due to the small number of activated carriers at room temperature, the NO_2_ gas-sensing ability can be improved six-fold by combining a-C with SnO_2_ (Fig. 5b). This is proof of the interfacial characteristics, where the energy equilibrium is changed due to the heterojunction. This tendency can further improve the sensing characteristics at 100 °C, 200 °C, and 300 °C, where there is a high carrier concentration at the interface. However, there are limits to increasing the interfacial reactivity to NO_2_ gas unlike in the case of conventional SnO_2_ surfaces (SI, Fig. S2a). In other words, the gas characteristics are not necessarily improved even with constantly high temperatures. As seen in Fig. 5b, if the temperature reaches a critical point between 200 °C and 300 °C, the a-C no longer increases the surface properties of SnO_2_, but can have other mechanisms. Therefore, the interfacial change according to the temperature dependence of a-C needs to be studied more accurately in the future. However, there is a difference between the SnO_2_/a-C characteristics that must be considered. In the analysis method of Fig. 3, the incident energy from the analytical tool passes through the interface between the a-C and the SnO_2_, whereas the gas-sensing reaction mechanism shown in Fig. 5b almost stops at the SnO_2_ interface. In other words, the role of a-C, which results in changes or improvements in the properties of the existing SnO_2_ materials, is the same, but the gas-sensing reaction mechanism is the most sensitive to surface reactions compared to other analytical methods. SI, Fig. S2b–e show the NO_2_ gas-sensing response and the response and recovery times at various temperatures for the SnO_2_/a-C sample. Compared with those of bare SnO_2_, the surface response sensitivities of SnO_2_/a-C are far superior, as expected, demonstrating excellent response time and recovery time in all temperature ranges. These representative gas-sensing indices before and after the a-C process is applied are presented in Supplementary Table 1.

As seen, instead of a crystalline shell, a-C can be deposited on a material to enhance its surface properties or to convert it into a new material. This can result in new functions in a short time, and it is expected to be widely used for industrial applications in the future because of its superior convenience and accessibility compared to other processes.

## Discussion

A coating method using a-C that can be easily and variously applied is suggested as a method of deposition on a base material. This method can improve the surface reactivity of existing materials while showing little of their physicochemical properties unlike existing heterogeneous junctions. Especially in the case of a-C, regardless of the surface state of the material, it is strongly adsorbed on the preformed material by controlling the process temperature or the process time. Only surface (or interface) characteristics of the existing material can be significantly enhanced. These surface sensitivities were measured using SEM, TEM, XRD, Raman, PL, XPS, and gas-sensing mechanisms. In particular, the NO_2_ gas-sensing ability of SnO_2_/a-C was almost six times better than that of bare-SnO_2_ even at room temperature. Therefore, if the a-C process parameters are controlled and the changes in each process condition are easily grasped at a glance, this advanced engineering technology can be applied to various industrial fields. This approach is also significant because it is a breakthrough that can be studied, without regard to materials and environment and without the need for expensive vacuum technology.

## Methods

As shown in Fig. 1, a home-made pyrotechnic device with a temperature range of 500–1200 °C was prepared for depositing a-C on a material. a-C was deposited onto thin films of an alumina substrate and as a shell coating of core SnO_2_ NWs. For the thin film, a spark was applied directly on the water, and a-C deposited on the water was drained and deposited on the alumina substrate. For the coating of the SnO_2_ NWs, the alumina substrate on which the SnO_2_ NWs were grown was used instead of a bare alumina substrate, while maintaining process methods such as thin film formation methods. The SnO_2_ NWs were produced using a thermal evaporation method with a Au catalyst at 900 °C for 1 h using 97% Ar and 3% O_2_ gases.

The morphologies of the SnO_2_/a-C core-shell structures were analysed using SEM (Hitachi S-4200) and TEM (200 kV, JEOL JEM-2010, Japan). HRTEM and SAED equipped with TEM and XRD (Philips X-pert MRD X-ray diffractometer) were used for microstructure analysis. The SnO_2_ and SnO_2_/a-C structures were analysed qualitatively and quantitatively via mapping and EDX with a TEM, and the chemical bonds on the surface were compared using Raman spectroscopy (LabRAM HR800, Jobin Yvon, France), PL (LabRAM HR800, Jobin Yvon, France), and XPS (K-Alpha plus, Thermo Fisher Scientific Inc., USA) analyses. In addition, the energy relationships of each component, such as the valence band maximum, work function, and Fermi level, were determined using UPS (Theta probe base system, Thermo Fisher Scientific Inc., USA). Gas sensing was performed with NO_2_, an oxidising gas. A semiconductor-based method in which the resistance changes depending on the degree of adsorption of gas was used for the gas-sensing experiment. The concentration of NO_2_ was fixed at 10 ppm, and the temperature range for sensing was from room temperature to 300 °C with 100 °C intervals.

## Supplementary information

Supplementary Information.

## Data Availability

All the data are available from the corresponding author on reasonable request.
