# Supplementary material for: Interface treatment using amorphous-carbon and its applications
Source: Sci Rep. 2020 Mar 5;10:4093. doi: 10.1038/s41598-020-61141-9 (PMC7058078; doi:10.1038/s41598-020-61141-9)
Supplement: Supplementary file 1 — Supplementary Information. [file 41598_2020_61141_MOESM1_ESM.docx]

**Supplementary information**

**Interface treatment using amorphous-carbon and its applications**

Myung Sik Choi^1^, Han Gil Na^1^, Jae Hoon Bang^1^, Sun-Woo Choi^2^, Sang Sub Kim^3^, Kyu Hyoung Lee^4^, Hyoun Woo Kim^1^* & Changhyun Jin^4^*

^1^Division of Materials Science and Engineering, Hanyang University, Seoul 04763, Republic of Korea

^2^Department of Materials Science and Engineering, Kangwon National University, Samcheok 25913, Republic of Korea

^3^Department of Materials Science and Engineering, Inha University, Incheon 402-751, Republic of Korea

^4^Department of Materials Science and Engineering, Yonsei University, Seoul, 03722, Republic of Korea

Myung Sik Choi and Han Gil Na had equal contribution as co-first authors.

*Corresponding author. Tel. +82 2 2220 0382. E-mail: hyounwoo@hanyang.ac.kr (H. W. Kim), Tel. +82 2 2123 2830. E-mail: chjin0910@gmail.com (C. Jin)

**Table S1.** **Comparison of 10-ppm NO_2_ gas-sensing characteristics of bare SnO_2_ and SnO_2_/a-C core-shell structures at various temperatures.**

|  | **SnO_2_** | | | **SnO_2_/a-C** | | |
| --- | --- | --- | --- | --- | --- | --- |
|  | Response  (R_g_/R_a_) | Response  time (s) | Recovery  time (s) | Response  (R_g_/R_a_) | Response  time (s) | Recovery  time (s) |
| **30^o^C** | 1.86 | 431 | >1000 | 6.56 | 267 | 614 |
| **100^o^C** | 3.44 | 334 | 997 | 66.42 | 95 | 117 |
| **200^o^C** | 9.21 | 123 | 225 | 97.7 | 21 | 15 |
| **300^o^C** | 5.29 | 110 | 25 | 51.14 | 64 | 10 |


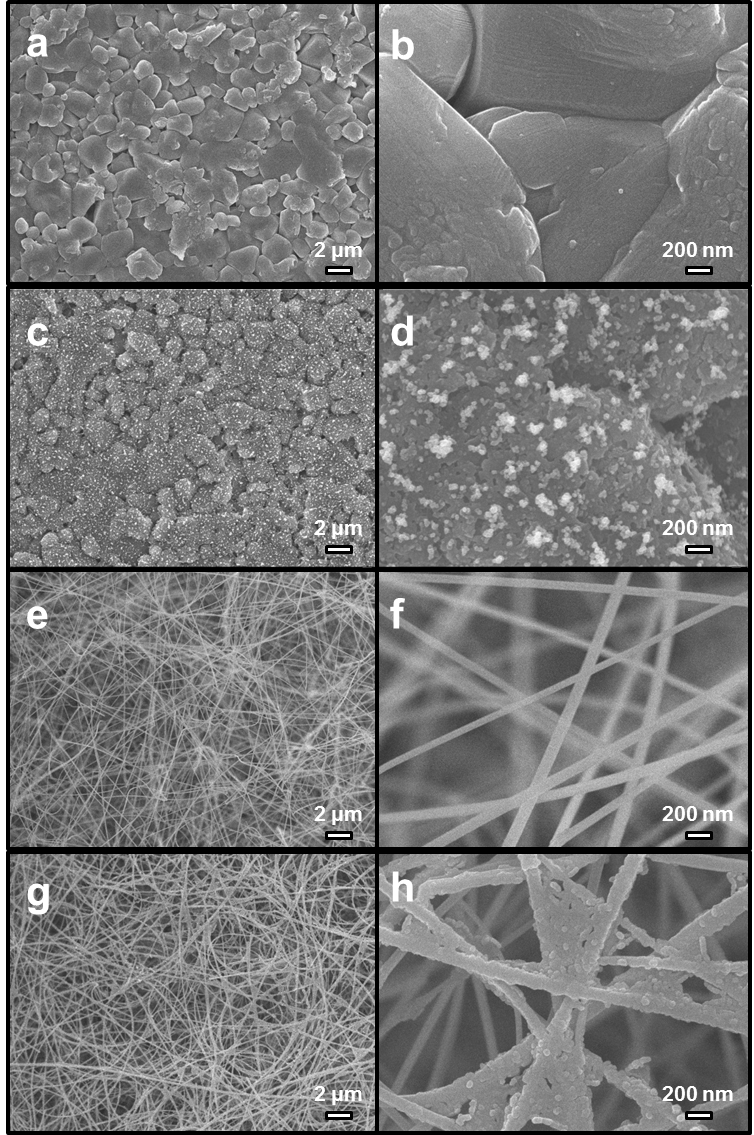


**Fig. S1. SEM images of a-C deposited on alumina substrate and SnO_2_ NWs.** Surface of alumina substrate at (a) low and (b) high magnification; surface of alumina substrate on which a-C is deposited at (c) low and (d) high magnification; SnO_2_ NWs at (e) low and (f) high magnification; SnO_2_ NWs on which a-C is deposited at (g) low and (h) high magnification.


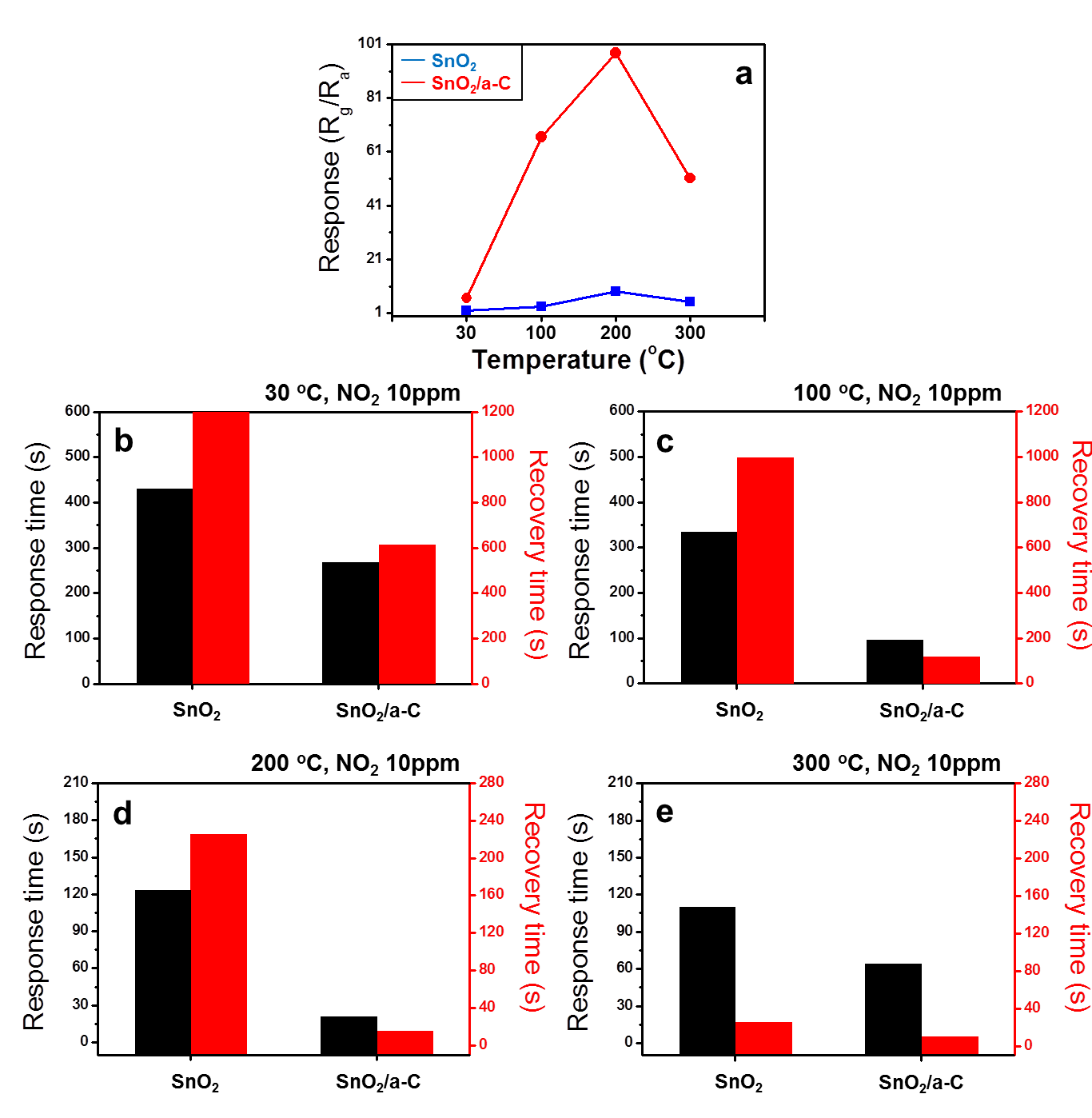


**Fig. S2. Comparison of gas-sensing characteristics under 10-ppm NO_2_ gas concentration with varying temperatures for bare SnO_2_ and SnO_2_/a-C core-shell structures.** (a) Temperature-dependent sensing response; response time and recovery time at (b) 30 °C, (c) 100 °C, (d) 200 °C, and (e) 300 °C.
